# Supplementary material for: Investigation of anxiety levels of 1637 healthcare workers during the epidemic of COVID-19
Source: PLoS One. 2020 Dec 22;15(12):e0243890. doi: 10.1371/journal.pone.0243890 (PMC7755185; doi:10.1371/journal.pone.0243890)
Supplement: Attachment — Submitted filename: Plos One Review 8.2020.docx [file pone.0243890.s006.docx]

This is an interesting article exploring anxiety levels and mental health interventions and support for healthcare workers from 5 hospitals in China.

Strengths include: large sample size, random selection of participants

There are several comments that need to be addressed:

-Is there any data on prevalence of depression or acute stress as this impacts anxiety levels and could interfere in interventions.

-Line 74/75: Can the authors explain whether the trained head nurse is a research coordinator or whether he/she has a clinical role? Also can authors provide further details about who checked the questionnaire for validity?

“The researcher explained 74 the purpose and the filling method of the questionnaire to the head nurses. The trained head 75 nurses distributed the electronic questionnaire to the nurses and guided them to fill the questionnaire.”

-Line 87, states: “The total anxiety score of healthcare workers in Zhejiang Province was 30.85 ± 6.89, which was higher than that of Chinese norm 9.78 ± 0.46 ^[4]^ 87 (t = 6.273, P < 0.01).”

Methodologically why are the authors comparing prevalence of anxiety of “Chinese norm” from an older reference in the Results section and running any comparison test? I presume it is reference 7 rather than 4 as this is mentioned in the Discussion Line 127/128: “This study showed that the score of anxiety level of healthcare workers in Zhejiang Province was higher than that of Chinese norm [7]”. This reference is discussing anxiety levels during the SARS outbreak in Beijing and uses other measures of psychological distress not assessed in the current study. Further please update the reference in Line 87 as it is incorrect: [4] Lehmann M, Bruenahl CA, Addo MM, Becker S, Schmiedel S, Lohse AW, et al. Acute 179 Ebola virus disease patient treatment and health-related quality of life in health care 180 professionals: A controlled study. J Psychosom Res. 2016; 83:69-74 is a study out of Germany and discusses Ebola so it’s unclear why this is being referenced here at all.

-Line 143/144 “Most nurses were women, facing the physiological period under protective clothing. Their emotions were easy to fluctuate.”

**This is a sexist statement and is unacceptable.**

-Was an intervention done? The abstract introduction is misleading and if no intervention was done this should be revised

“This study aimed to investigate the anxiety levels of healthcare workers and to **provide effective and accurate social and psychological interventions** for healthcare workers during the epidemic of COVID-19 in Zhejiang Province, China.”

-The discussion section abruptly ends and needs to be revised for cohesion.
